# Optimal Cutoff Titers for Indirect Immunofluorescence Assay for Diagnosis of Scrub Typhus

**DOI:** 10.1128/JCM.01680-15

**Published:** 2015-10-16

**Authors:** Cherry Lim, Stuart D. Blacksell, Achara Laongnualpanich, Pacharee Kantipong, Nicholas P. J. Day, Daniel H. Paris, Direk Limmathurotsakul

**Affiliations:** a Mahidol-Oxford Tropical Medicine Research Unit, Faculty of Tropical Medicine, Mahidol University, Bangkok, Thailand; b Centre for Tropical Medicine and Global Health, Nuffield Department of Clinical Medicine, University of Oxford, Oxford, United Kingdom; c Chiang Rai Prachanukroh Hospital, Chiang Rai, Thailand; d Department of Tropical Hygiene, Faculty of Tropical Medicine, Mahidol University, Bangkok, Thailand

## Abstract

We determined the optimal cutoff titers in admission and convalescent-phase samples for scrub typhus indirect immunofluorescence assay using Bayesian latent class models. Cutoff titers of ≥1:3,200 in an admission sample or of a ≥4-fold rise to ≥1:3,200 in a convalescent-phase sample provided the highest accuracy (sensitivity, 81.6%; specificity, 100%).

## TEXT

Scrub typhus is an infectious disease caused by the Gram-negative intracellular bacterium Orientia tsutsugamushi (1). Indirect immunofluorescence assay (IFA) is considered a serological reference test for the disease; however, the cutoff antibody titer to use remains controversial (2). Conventional cutoff titers for the IFA IgM of ≥1:400 in an admission sample or of a ≥4-fold rise to ≥1:200 in a convalescent-phase sample were first proposed by Brown et al. in 1983 (3) and have been regularly used since then (4). However, the IFA IgM has long been suspected of having a high rate of false positivity (5). In 2011, we proposed the scrub typhus infection criteria (STIC) (a combination of culture, PCR assays, and IFA IgM), in which IFA IgM cutoff titers of either ≥1:12,800 in an admission sample or of a ≥4-fold rise to ≥1:200 in a convalescent-phase sample were used (6). The high cutoff titer of IFA IgM for the admission sample (≥1:12,800) was proposed to provide a robust diagnosis of acute scrub typhus infection in research settings (5), and the STIC have been used as a comparator to evaluate the accuracy of alternative diagnostic tests (6, 7). Using Bayesian latent class models (LCMs), we recently showed that IFA IgM with cutoff titers defined by the STIC in paired samples had low sensitivity and specificity (70.0% and 83.8%, respectively) (8). Here, we reevaluated the performance of IFA IgM by selecting optimal cutoff titers using Bayesian LCMs and generating unbiased receiver operating characteristic (ROC) curves.

The data set used in this study was from a prospective study of acute undifferentiated febrile illness in an area where scrub typhus is endemic, Chiang Rai, Northern Thailand (6, 7). In brief, adult patients presenting with acute febrile illness of <2-weeks duration with three negative malaria blood smears and no evidence of a primary focus of infection were enrolled. Every patient was examined for the presence of an eschar. Admission blood samples were tested using an *in vitro* culture for Orientia tsutsugamushi, a nested 56-kDa PCR assay, a 47-kDa-based real-time PCR assay, a *groEL*-based real-time PCR assay, an IgM-based IFA, and an IgM-based rapid immunochromatographic test (PanBio ICT, Australia) (6, 7). Convalescent-phase blood samples were also tested using IFA IgM. Antigens used for IFA were pooled from Orientia tsutsugamushi Karp, Kato, and Gilliam strains as described previously (6, 7). The IFA results were expressed as the highest serum dilution in which O. tsutsugamushi-specific IgM was detected, ranging from ≤1:50 (negative), 1:100, 1:200, and at a 2-fold dilution series up to a maximum of ≥1:25,600 (6, 7). Ethical approval for the prospective study was obtained from the ethical committees of Chiang Rai Hospital, the Ministry of Public Health, Thailand, and from the Oxford Tropical Research Ethics Committee, United Kingdom. Signed written inform consent was obtained from every subject enrolled into the study (6, 7).

To determine the optimal cutoff titers in an admission sample and a convalescent-phase sample for IFA IgM, two stages of statistical analysis were performed. First, we used Bayesian LCMs to generate unbiased ROC curves for the sensitivities and specificities of all possible cutoff titers of IFA IgM in the admission sample alone. Bayesian LCMs with conditional dependence between IFA IgM and an IgM immunochromatographic test (ICT) were used as previously described (8). In brief, Bayesian LCMs estimated the prevalence and the sensitivity and specificity of each diagnostic test with their 95% credible intervals (CrI) using the Markov chain Monte Carlo (MCMC) method (8). Bayesian LCMs do not assume that any diagnostic test or combination of diagnostic tests is perfect. The true disease status of each patient was estimated by the model in each MCMC iteration and expressed as the overall disease prevalence (8). Unbiased ROC curves were generated as previously described (9). Potential optimal cutoff titers for admission IFA IgM were selected based on the overall accuracy at each titer. Second, we used Bayesian LCMs to generate unbiased ROC curves for the sensitivities and specificities of IFA IgM using a combination of each potential optimal cutoff titer in the admission sample selected in the first stage and a ≥4-fold rise to all possible cutoff titers of IFA IgM in the convalescent-phase sample. The optimal cutoff titers for the IFA IgM were selected based on overall accuracy. Text S1 and S2 in the supplemental material provide full data sets and the models used, respectively.

A total of 161 patients were included in the study. The median age was 41 years (interquartile range [IQR], 29 to 51 years), and 63% (99/158) were male. The median duration of fever before admission to hospital was 5 days (IQR, 3 to 7 days), and 138 patients (85.7%) had convalescent-phase samples available. The median duration between the admission sample and convalescent-phase sample was 12 days (IQR, 3 to 14 days). Culture, a combination of PCR assays, IgM ICT, and the presence of eschar were positive in 9, 27, 31, and 17 patients, respectively. Overall, 62 patients were considered IFA IgM positive based on the conventional cutoff titers (42 had an admission IFA IgM of ≥1:400), and 46 patients were considered IFA IgM positive based on STIC (20 had admission IFA IgM of ≥1:12,800).

**FIG 1:**
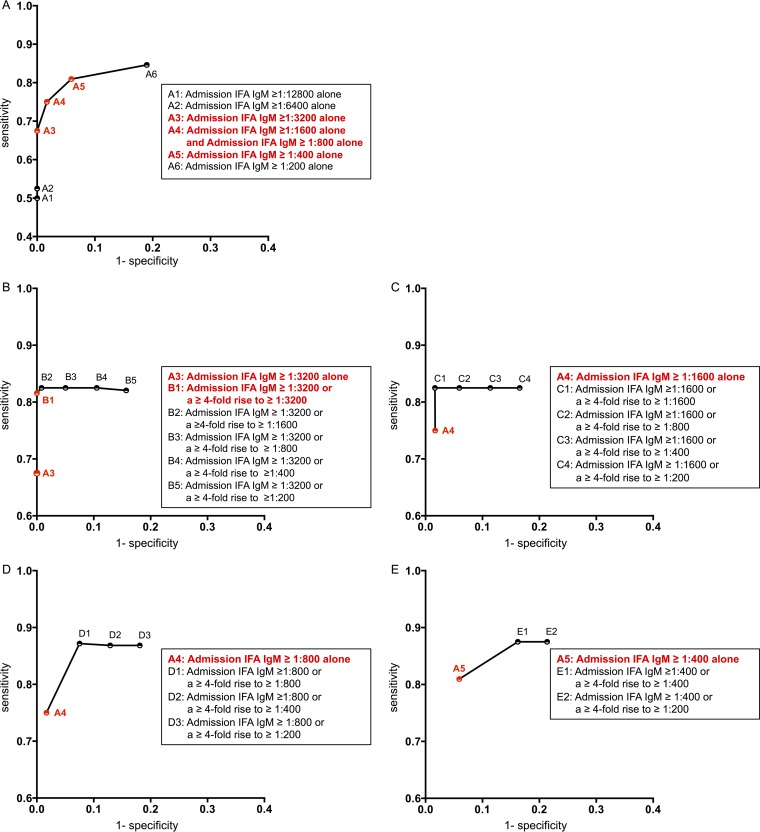
Unbiased receiver operating characteristic (ROC) curves using Bayesian latent class models (LCMs) for all possible cutoff titers of admission IFA IgM in an admission sample alone (A) and for IFA IgM of either ≥1:3,200 (B), ≥1:1,600 (C), ≥1:800 (D), and ≥1:400 (E) in an admission sample or a ≥4-fold rise to various cutoff titers in a convalescent-phase sample.

First, we used the unbiased ROC curves to evaluate the performance of admission IFA IgM in an admission sample alone (Fig. 1A) and showed that an admission IFA IgM of ≥1:3,200 had 67.5% sensitivity and 100% specificity, while an admission IFA IgM of ≥1:400 had a higher sensitivity of 81.0% but a lower specificity of 94.1%. Therefore, a range of on-admission cutoff titers from ≥1:400 to ≥1:3,200 was evaluated further. Second, we used unbiased ROC curves to evaluate different cutoff titers of IFA IgM in paired samples (Fig. 1B to E). We found that adding a criterion of a ≥4-fold rise to ≥1:200 in a convalescent-phase sample to the diagnostic criteria lowered the overall specificities of IFA IgM. For example, adding the criterion of a ≥4-fold rise to ≥1:200 in the convalescent-phase sample to the conventional cutoff led to a decrease of specificity from 94.1% to 78.6% (Fig. 1E). Similarly, adding the criterion of a ≥4-fold rise to ≥1:200 in the convalescent-phase sample to the cutoff defined by STIC led to a decrease of specificity from 100.0% to 83.8% (data not shown). Cutoff titers of ≥1:3,200 in the admission sample or a ≥4-fold rise to ≥1:3,200 in the convalescent-phase sample had the highest accuracy with a sensitivity of 81.6% (95% CrI, 70.5 to 91.2) and a specificity of 100% (95% CrI, 98.4 to 100), and these values were chosen as the optimal cutoff titers for the IFA IgM.

As we found that a criterion of a ≥4-fold rise to ≥1:200 in a convalescent-phase sample led to the low specificities of conventional and STIC cutoff titers of IFA IgM, we validated this finding by evaluating the results of IFA IgM in those who had alternative diagnoses in our data set as previously described (7). We found that 4 murine typhus patients and 4 dengue patients who had negative results in culture, PCR assays, and presence of eschar for scrub typhus each had a 4-fold rise of IFA IgM to 1:200, 1:400, or 1:800 in the convalescent-phase samples.

In this study, we used an unbiased Bayesian LCM approach and defined optimal cutoff titers for IFA IgM as either ≥1:3,200 in an admission samples or as a ≥4-fold rise to ≥1:3,200 in a convalescent-phase sample. With these new cutoff titers, the sensitivity and specificity of the IFA IgM is 81.6% and 100%, respectively, indicating that they can be used to diagnose scrub typhus with high confidence in clinical and research settings. The low specificity caused by the low cutoff titers of IFA IgM could be due to cross-reactivity of the IFA IgM with infections caused by other organisms in our setting, and this is also likely to occur in other tropical countries (9). Although Brown et al. reported a high specificity for the IFA IgM using the conventional cutoff in the convalescent-phase sample, the median duration between the admission sample and the convalescent-phase sample in non-scrub typhus control patients in his study was unknown (3). Based on our findings, the capping titer for IFA IgM in our setting could be ≥1:6,400 so that it can diagnose acute scrub typhus in a patient who presents with IFA IgM 1:1,600 on admission with a 4-fold rise to ≥1:6,400 in a convalescent-phase sample. As the accuracy and cost-benefit of diagnostic tests could vary based on prevalence, clinical variability, and availability and timing of convalescent-phase samples (10, 11), further studies to evaluate optimal cutoff titers, accuracy, and benefits of IFA IgM in different settings are still required.

The biased selection of cutoff titers is a common problem affecting many serological tests for tropical infectious diseases because the reference tests are rarely, if ever, perfect (12). In addition, many serological tests for tropical infectious diseases, including the scrub typhus IFA IgM, are still based on the judgement of readers; therefore, these tests remain inherently subjective (1). Future development of new serological diagnostic tests for tropical infectious diseases should be based on methodologies with objective readouts such as enzyme-linked immunosorbent assay (ELISA), and the optimal cutoffs in admission and convalescent-phase samples should be selected based on appropriate statistical models.

## Supplementary Material

Supplemental material
