# Supplementary material for: Optimal Cutoff Titers for Indirect Immunofluorescence Assay for Diagnosis of Scrub Typhus
Source: J Clin Microbiol. 2015 Oct 16;53(11):3663–6. doi: 10.1128/JCM.01680-15 (PMC4609688; doi:10.1128/JCM.01680-15)
Supplement: Supplemental material [file supp_53_11_3663__index.html]

Optimal Cutoff Titers for Indirect Immunofluorescence Assay for Diagnosis of Scrub Typhus — Supplemental material 

# Optimal Cutoff Titers for Indirect Immunofluorescence Assay for Diagnosis of Scrub Typhus

## Supplemental material

- Supplemental file 1 -

  Text S1 (Data set for Bayesian latent-class models)

  PDF, 93K
- Supplemental file 2 -

  Text S2 (WinBUGS models)

  PDF, 67K
