# Supplementary material for: Optimal Cutoff Titers for Indirect Immunofluorescence Assay for Diagnosis of Scrub Typhus
Source: J Clin Microbiol. 2015 Oct 16;53(11):3663–6. doi: 10.1128/JCM.01680-15 (PMC4609688; doi:10.1128/JCM.01680-15)
Supplement: Supplemental material [file JCM.01680-15_zjm999094589so1.pdf]

Text S1. The data set for Bayesian latent class models.

The following is the complete data set for Bayesian latent class model to estimate the accuracies of diagnostic tests for scrub typhus with conditional dependence between IFA IgM and PanBio IgM ICT. The first, second, third, fourth, and fifth columns are the results of *in vitro* isolation of *O. tsutsugamushi* (culture), a combination of PCR assays, STIC-based IFA IgM (positive when IFA IgM in admission sample was ≥1:12,800 and/or ≥4-fold rise to ≥1:200 in convalescent sample compared to admission sample), PanBio IgM ICT and presence of eschar, respectively. 1= positive result and 0= negative result. Each row represents one independent participant. The sixth and seventh columns are the highest IgM titres detected by IFA IgM in admission and convalescent sample, respectively. The raw data on IgM titres were converted into binary data for each possible cutoff titre used in the model.

```
# Data
list(y=structure(
.Data=c(
     0,      1,      1,      1,      1,   400, 25600,
     0,      0,      0,      0,      0,    50,    50,
     0,      0,      1,      1,      1, 25600, 25600,
     0,      0,      0,      1,      1,  3200,  3200,
     0,      0,      1,      1,      1, 25600, 25600,
     0,      0,      0,      0,      0,    50,    NA,
     0,      0,      1,      1,      0, 25600, 25600,
     1,      1,      1,      1,      1, 25600,    NA,
     0,      0,      0,      0,      0,   100,    50,
     0,      0,      0,      0,      0,    50,    50,
     0,      0,      0,      1,      0,  3200,  3200,
     0,      0,      0,      1,      0,   800,   400,
     0,      1,      1,      1,      0, 12800,    NA,
     1,      1,      1,      1,      1, 25600, 25600,
     0,      0,      0,      0,      0,    50,   100,
     0,      1,      0,      1,      0,  3200,  3200,
     0,      1,      0,      0,      0,    50,    50,
     0,      0,      0,      0,      0,  1600,   800,
     0,      0,      0,      0,      0,   100,    NA,
     0,      0,      1,      0,      0,    50,   200,
     0,      0,      0,      0,      0,   100,    50,
     0,      0,      0,      0,      0,   100,   100,
     1,      0,      0,      0,      0,    50,   100,
     0,      0,      0,      0,      0,    50,    50,
     0,      0,      0,      0,      0,    50,    50,
     0,      0,      0,      0,      0,    50,    50,
     0,      0,      1,      0,      0,   100,   800,
     0,      0,      0,      0,      0,    50,    50,
     0,      0,      0,      0,      0,    50,    NA,
     0,      0,      0,      0,      0,    50,    50,
     0,      1,      0,      1,      0,  3200,    NA,
     0,      0,      0,      0,      0,    50,    NA,
     0,      0,      0,      0,      0,   200,    50,
     0,      1,      1,      0,      0, 25600,    50,
     1,      1,      1,      1,      1, 25600,  1600,
     0,      0,      0,      0,      0,    50,   100,
     0,      1,      1,      0,      0, 25600, 25600,
     0,      0,      0,      0,      0,    50,   100,
     0,      0,      0,      0,      0,   100,   100,
     0,      0,      0,      0,      0,   100,   100,
     0,      0,      1,      1,      1, 12800,    NA,
     0,      0,      0,      0,      0,   200,    NA,
     0,      0,      0,      0,      0,    50,    50,
     0,      0,      1,      0,      0,   100,   400,
     0,      0,      0,      0,      0,   400,   200,
     0,      1,      0,      1,      1,  3200,    NA,
     0,      0,      0,      0,      0,    50,    50,
     0,      0,      0,      0,      0,   200,   400,
     0,      0,      0,      0,      0,   200,    50,
     0,      0,      0,      0,      0,   200,   200,
     0,      0,      0,      0,      1,    50,    50,
     1,      0,      0,      0,      0,    50,    50,
```

```
0,     0,     1,     1,     0, 12800,    NA,
0,     0,     0,     0,     0,    50,    50,
0,     0,     0,     0,     0,    50,    50,
1,     1,     1,     1,     1, 25600, 25600,
0,     0,     0,     0,     0,   100,    NA,
0,     0,     0,     0,     0,    50,    50,
0,     0,     0,     0,     0,    50,    50,
0,     0,     0,     0,     0,    50,    50,
0,     0,     0,     0,     0,   200,   200,
0,     0,     0,     0,     0,   100,   200,
0,     0,     0,     0,     0,   800,   800,
0,     0,     0,     0,     0,    50,    NA,
0,     0,     0,     0,     0,    50,    50,
0,     0,     0,     0,     0,    50,   100,
0,     0,     0,     0,     0,    50,    NA,
0,     0,     0,     0,     0,   200,   400,
0,     0,     0,     0,     0,   100,   100,
0,     1,     1,     1,     0, 25600, 25600,
0,     0,     0,     0,     0,    50,    50,
0,     0,     0,     0,     0,   100,    NA,
0,     0,     0,     0,     0,   100,    50,
0,     1,     1,     1,     0,   100, 25600,
0,     0,     0,     0,     0,    50,   100,
0,     0,     0,     0,     0,    50,    50,
0,     0,     0,     0,     0,   800,   800,
0,     0,     1,     0,     0,   400, 12800,
0,     0,     0,     0,     0,   200,   200,
0,     1,     1,     1,     0, 25600, 25600,
0,     0,     0,     0,     0,   100,   100,
0,     0,     0,     0,     0,    50,    NA,
0,     1,     0,     0,     0,    50,    50,
0,     0,     0,     0,     0,    50,    50,
0,     0,     0,     0,     0,   100,   200,
0,     0,     1,     0,     0,   100,   400,
0,     0,     0,     0,     0,    50,    NA,
0,     0,     0,     0,     0,   400,   200,
0,     0,     0,     0,     0,    50,   100,
0,     0,     1,     0,     0,    50,   200,
0,     0,     0,     0,     0,    50,    NA,
0,     0,     0,     0,     0,    50,    50,
0,     1,     1,     1,     1, 25600, 25600,
0,     0,     1,     1,     0, 25600, 25600,
0,     0,     1,     0,     0,   100,   400,
0,     0,     0,     0,     0,   400,   400,
0,     0,     0,     0,     0,    50,    50,
0,     0,     0,     0,     0,    50,    50,
0,     1,     1,     1,     0, 12800,    NA,
0,     0,     1,     0,     0,    50,   400,
0,     0,     1,     0,     0,    50,   400,
0,     0,     1,     0,     0,    50,   200,
0,     0,     0,     0,     0,    50,   100,
0,     1,     1,     0,     0,   400,  1600,
0,     0,     0,     0,     0,    50,    NA,
0,     0,     1,     0,     0,    50,   200,
0,     0,     1,     0,     0,   200,   800,
0,     0,     0,     0,     0,    50,    50,
0,     0,     1,     0,     0,    50,   800,
0,     0,     0,     0,     0,   100,   100,
0,     1,     0,     0,     0,    50,    50,
0,     0,     0,     0,     0,    50,    50,
1,     1,     1,     0,     0,   200, 25600,
0,     0,     0,     0,     0,    50,    50,
0,     0,     0,     0,     0,   100,   200,
0,     0,     0,     0,     0,    50,    NA,
0,     0,     0,     0,     0,    50,   100,
0,     0,     0,     0,     0,   100,   100,
0,     0,     0,     0,     0,   100,    50,
0,     0,     0,     0,     0,    50,    50,
0,     0,     0,     0,     0,    50,    50,
0,     0,     1,     0,     0,    50,   800,
0,     0,     1,     0,     0,    50,  1600,
0,     0,     1,     0,     0,   100,   400,
0,     1,     1,     1,     1,  3200, 25600,
0,     0,     0,     0,     0,    50,    50,
0,     0,     0,     0,     0,    50,    NA,
0,     0,     0,     0,     0,    50,    50,
0,     0,     0,     0,     0,   200,   400,
0,     0,     0,     0,     0,    50,    50,
0,     0,     0,     0,     0,    50,    50,
```

```
      0,      0,      0,      0,      0,    50,    50,
      0,      0,      0,      0,      0,   200,   100,
      0,      0,      0,      0,      0,   200,   200,
      1,      1,      1,      1,      0, 25600, 25600,
      1,      0,      0,      0,      0,   800,   800,
      0,      0,      0,      0,      0,    50,    50,
      0,      0,      0,      0,      0,   200,   200,
      0,      1,      1,      1,      1,   800, 25600,
      0,      0,      0,      0,      0,    50,    50,
      0,      0,      0,      0,      0,   200,    50,
      0,      0,      0,      0,      0,    50,    50,
      0,      0,      0,      0,      0,    50,    50,
      0,      0,      1,      0,      0,    50,   200,
      0,      0,      1,      0,      0,    50,   800,
      0,      0,      0,      0,      0,    50,    50,
      0,      1,      1,      1,      1, 12800,  3200,
      0,      1,      1,      1,      0,  6400, 25600,
      0,      1,      1,      1,      1, 25600, 25600,
      0,      0,      0,      0,      0,    50,    50,
      0,      0,      0,      0,      1,   100,   100,
      0,      0,      0,      0,      0,    50,    50,
      0,      0,      1,      0,      0,    50,   200,
      0,      0,      0,      0,      0,    50,    50,
      0,      0,      0,      0,      0,   200,    NA,
      0,      0,      0,      1,      0,   400,   100,
      0,      0,      0,      0,      0,    50,    50,
      0,      0,      0,      0,      0,    50,    50,
      0,      0,      0,      1,      0,   400,   800,
      0,      0,      0,      0,      0,    50,    50
),.Dim=c(161,5)),
pattern=structure(
.Data=c(
       1,      1,      1,      1,      1,
       1,      1,      1,      1,      0,
       1,      1,      1,      0,      1,
       1,      1,      0,      1,      1,
       1,      0,      1,      1,      1,
       0,      1,      1,      1,      1,
       1,      1,      1,      0,      0,
       1,      1,      0,      1,      0,
       1,      0,      1,      1,      0,
       0,      1,      1,      1,      0,
       1,      1,      0,      0,      1,
       1,      0,      1,      0,      1,
       0,      1,      1,      0,      1,
       1,      0,      0,      1,      1,
       0,      1,      0,      1,      1,
       0,      0,      1,      1,      1,
       1,      1,      0,      0,      0,
       1,      0,      1,      0,      0,
       1,      0,      0,      1,      0,
       1,      0,      0,      0,      1,
       0,      1,      1,      0,      0,
       0,      1,      0,      1,      0,
       0,      1,      0,      0,      1,
       0,      0,      1,      1,      0,
       0,      0,      1,      0,      1,
       0,      0,      0,      1,      1,
       1,      0,      0,      0,      0,
       0,      1,      0,      0,      0,
       0,      0,      1,      0,      0,
       0,      0,      0,      1,      0,
       0,      0,      0,      0,      1,
       0,      0,      0,      0,      0
),.Dim=c(32,5)))
```

The following is an example dataset for Bayesian latent class model to search for the optimal cutoff titre for IFA IgM. The first, second, third, fourth, and fifth columns are the results of *in vitro* isolation of *O. tsutsugamushi* (culture), a combination of PCR assays, IFA IgM (positive when IFA IgM in admission sample was ≥1:3,200 and/or ≥4-fold rise to ≥1:3,200 in convalescent sample compared to admission sample), PanBio IgM ICT and presence of eschar, respectively. 1= positive result and 0= negative result. Each row represents one independent participant.

```
## Data
list(y=structure(
.Data=c(
     0,     0,     0,     0,     0,
     0,     1,     1,     1,     1,
     0,     0,     0,     0,     0,
     0,     0,     1,     1,     1,
     0,     0,     1,     1,     1,
     0,     0,     1,     1,     1,
     0,     0,     0,     0,     0,
     0,     0,     1,     1,     0,
     1,     1,     1,     1,     1,
     0,     0,     0,     0,     0,
     0,     0,     0,     0,     0,
     0,     0,     1,     1,     0,
     0,     0,     0,     1,     0,
     0,     1,     1,     1,     0,
     1,     1,     1,     1,     1,
     0,     0,     0,     0,     0,
     0,     1,     1,     1,     0,
     0,     1,     0,     0,     0,
     0,     0,     0,     0,     0,
     0,     0,     0,     0,     0,
     0,     0,     0,     0,     0,
     0,     0,     0,     0,     0,
     0,     0,     0,     0,     0,
     1,     0,     0,     0,     0,
     0,     0,     0,     0,     0,
     0,     0,     0,     0,     0,
     0,     0,     0,     0,     0,
     0,     0,     0,     0,     0,
     0,     0,     0,     0,     0,
     0,     0,     0,     0,     0,
     0,     0,     0,     0,     0,
     0,     1,     1,     1,     0,
     0,     0,     0,     0,     0,
     0,     0,     0,     0,     0,
     0,     1,     1,     0,     0,
     1,     1,     1,     1,     1,
     0,     0,     0,     0,     0,
     0,     1,     1,     0,     0,
     0,     0,     0,     0,     0,
     0,     0,     0,     0,     0,
     0,     0,     0,     0,     0,
     0,     0,     1,     1,     1,
     0,     0,     0,     0,     0,
     0,     0,     0,     0,     0,
     0,     0,     0,     0,     0,
     0,     0,     0,     0,     0,
     0,     1,     1,     1,     1,
     0,     0,     0,     0,     0,
     0,     0,     0,     0,     0,
     0,     0,     0,     0,     0,
     0,     0,     0,     0,     0,
     0,     0,     0,     0,     1,
     1,     0,     0,     0,     0,
     0,     0,     1,     1,     0,
     0,     0,     0,     0,     0,
     0,     0,     0,     0,     0,
     1,     1,     1,     1,     1,
     0,     0,     0,     0,     0,
     0,     0,     0,     0,     0,
     0,     0,     0,     0,     0,
```

```
0,      0,      0,      0,      0,
0,      0,      0,      0,      0,
0,      0,      0,      0,      0,
0,      0,      0,      0,      0,
0,      0,      0,      0,      0,
0,      0,      0,      0,      0,
0,      0,      0,      0,      0,
0,      0,      0,      0,      0,
0,      0,      0,      0,      0,
0,      0,      0,      0,      0,
0,      1,      1,      1,      0,
0,      0,      0,      0,      0,
0,      0,      0,      0,      0,
0,      0,      0,      0,      0,
0,      1,      1,      1,      0,
0,      0,      0,      0,      0,
0,      0,      0,      0,      0,
0,      0,      0,      0,      0,
0,      0,      1,      0,      0,
0,      0,      0,      0,      0,
0,      1,      1,      1,      0,
0,      0,      0,      0,      0,
0,      0,      0,      0,      0,
0,      1,      0,      0,      0,
0,      0,      0,      0,      0,
0,      0,      0,      0,      0,
0,      0,      0,      0,      0,
0,      0,      0,      0,      0,
0,      0,      0,      0,      0,
0,      0,      0,      0,      0,
0,      0,      0,      0,      0,
0,      0,      0,      0,      0,
0,      0,      0,      0,      0,
0,      1,      1,      1,      1,
0,      0,      1,      1,      0,
0,      0,      0,      0,      0,
0,      0,      0,      0,      0,
0,      0,      0,      0,      0,
0,      0,      0,      0,      0,
0,      1,      1,      1,      0,
0,      0,      0,      0,      0,
0,      0,      0,      0,      0,
0,      0,      0,      0,      0,
0,      0,      0,      0,      0,
0,      1,      0,      0,      0,
0,      0,      0,      0,      0,
0,      0,      0,      0,      0,
0,      0,      0,      0,      0,
0,      0,      0,      0,      0,
0,      0,      0,      0,      0,
0,      0,      0,      0,      0,
0,      1,      0,      0,      0,
0,      0,      0,      0,      0,
1,      1,      1,      0,      0,
0,      0,      0,      0,      0,
0,      0,      0,      0,      0,
0,      0,      0,      0,      0,
0,      0,      0,      0,      0,
0,      0,      0,      0,      0,
0,      0,      0,      0,      0,
0,      0,      0,      0,      0,
0,      0,      0,      0,      0,
0,      0,      0,      0,      0,
0,      0,      0,      0,      0,
0,      0,      0,      0,      0,
0,      1,      1,      1,      1,
0,      0,      0,      0,      0,
0,      0,      0,      0,      0,
0,      0,      0,      0,      0,
0,      0,      0,      0,      0,
0,      0,      0,      0,      0,
0,      0,      0,      0,      0,
0,      0,      0,      0,      0,
0,      0,      0,      0,      0,
0,      0,      0,      0,      0,
1,      1,      1,      1,      0,
1,      0,      0,      0,      0,
0,      0,      0,      0,      0,
0,      0,      0,      0,      0,
```

```
     0,      1,      1,      1,      1,
     0,      0,      0,      0,      0,
     0,      0,      0,      0,      0,
     0,      0,      0,      0,      0,
     0,      0,      0,      0,      0,
     0,      0,      0,      0,      0,
     0,      0,      0,      0,      0,
     0,      0,      0,      0,      0,
     0,      1,      1,      1,      1,
     0,      1,      1,      1,      0,
     0,      1,      1,      1,      1,
     0,      0,      0,      0,      0,
     0,      0,      0,      0,      1,
     0,      0,      0,      0,      0,
     0,      0,      0,      0,      0,
     0,      0,      0,      0,      0,
     0,      0,      0,      0,      0,
     0,      0,      0,      1,      0,
     0,      0,      0,      0,      0,
     0,      0,      0,      0,      0,
     0,      0,      0,      1,      0,
     0,      0,      0,      0,      0
),.Dim=c(161,5)),
pattern=structure(
.Data=c(
        1,      1,      1,      1,      1,
        1,      1,      1,      1,      0,
        1,      1,      1,      0,      1,
        1,      1,      0,      1,      1,
        1,      0,      1,      1,      1,
        0,      1,      1,      1,      1,
        1,      1,      1,      0,      0,
        1,      1,      0,      1,      0,
        1,      0,      1,      1,      0,
        0,      1,      1,      1,      0,
        1,      1,      0,      0,      1,
        1,      0,      1,      0,      1,
        0,      1,      1,      0,      1,
        1,      0,      0,      1,      1,
        0,      1,      0,      1,      1,
        0,      0,      1,      1,      1,
        1,      1,      0,      0,      0,
        1,      0,      1,      0,      0,
        1,      0,      0,      1,      0,
        1,      0,      0,      0,      1,
        0,      1,      1,      0,      0,
        0,      1,      0,      1,      0,
        0,      1,      0,      0,      1,
        0,      0,      1,      1,      0,
        0,      0,      1,      0,      1,
        0,      0,      0,      1,      1,
        1,      0,      0,      0,      0,
        0,      1,      0,      0,      0,
        0,      0,      1,      0,      0,
        0,      0,      0,      1,      0,
        0,      0,      0,      0,      1,
        0,      0,      0,      0,      0
),.Dim=c(32,5)))
```
