# Supplementary material for: Optimal Cutoff Titers for Indirect Immunofluorescence Assay for Diagnosis of Scrub Typhus
Source: J Clin Microbiol. 2015 Oct 16;53(11):3663–6. doi: 10.1128/JCM.01680-15 (PMC4609688; doi:10.1128/JCM.01680-15)
Supplement: Supplemental material [file JCM.01680-15_zjm999094589so2.pdf]

**Text S2. WinBUGS models.**

The following is the WinBUGS codes for Bayesian latent-class model with conditional dependence between results of IFA IgM and PanBio IgM ICT. The same model structure is applied to each and every dataset created

```
model{

for (i in 1:161){
    status[i]~dbern(prev)

###### Likellihood #######
for (j in 1:5){
    y[i,j]~dbern(p[i,j])
    ypred[i,j]~dbern(p[i,j])
    }
    }

for (i in 1:161){
for (j in 1:2){
    logit(p[i,j])<-status[i]*alpha[j]+(1-status[i])*beta[j]
}
for (j in 3:4){
    logit(p[i,j])<-status[i]*alpha[j]+(1-status[i])*beta[j]+status[i]*re[i]*IgM
    logit(pstatus[i,j])<-status[i]*alpha[j]+status[i]*re[i]*IgM+(1-status[i])*(-1000)
}
    logit(p[i,5])<-status[i]*alpha[5]+(1-status[i])*beta[5]
    re[i]~dnorm(0,1)
}

###### Prior ######
prev~dbeta(0.5,0.5)
IgM~dnorm(0.0,0.1)I(0,6)
alpha[1]~dnorm(0.0,0.1)
 logit(s[1])<-alpha[1]
beta[1]<--1000
for (j in 2:5){
        alpha[j]~dnorm(0.0,0.1)
        beta[j]~dnorm(0.0,0.1)I(,1)
        logit(s[j])<-alpha[j]
        logit(x[j])<--beta[j]
    }
se[1]<-s[1]
se[2]<-s[2]
se[5]<-s[5]
for (j in 3:4){
        se[j]<-sum(pstatus[,j])/sum(status[])
}

#### Unbiased estimation of sensitivity, specificity and accuracy #####
for (i in 1:161){
for (j in 1:5){
        diseasetestpos[i,j]<-status[i]*y[i,j]
        nondiseasetestneg[i,j]<-(1-status[i])*(1-y[i,j])
}
}
for (j in 1:5){
        sen[j]<-sum(diseasetestpos[,j])/sum(status[])
        spec[j]<-sum(nondiseasetestneg[,j])/(161-sum(status[]))
}

for (j in 1:5){
accuracy[j]<-(sum(diseasetestpos[,j])+ sum(nondiseasetestneg[,j]))/161
}

###### Prediction ######
for (i in 1:161){
for (k in 1:32){
for (j in 1:5){
    arraymatched[i,k,j]<-equals(ypred[i,j],pattern[k,j])
    }
    nmatched[i,k]<-sum(arraymatched[i,k,])
    matchedpattern[i,k]<-equals(nmatched[i,k],5)
    }
    }
```

```
for (k in 1:32){
    freqpred[k]<-sum(matchedpattern[,k])
}

####### Bayesian p value ########
for (i in 1:161){
for (k in 1:32){
for (j in 1:5){
    arraymatchedobs[i,k,j]<-equals(y[i,j],pattern[k,j])
    }
    nmatchedobs[i,k]<-sum(arraymatchedobs[i,k,])
    matchedpatternobs[i,k]<-equals(nmatchedobs[i,k],5)
    }
    }
for (k in 1:32){
    freqobs[k]<-sum(matchedpatternobs[,k])
}
for (k in 1:32){
pvalue[k]<-step(freqpred[k]-freqobs[k])
}
}
```
